# A Comparative Study of Traffic Classification Techniques for Smart City Networks

**DOI:** 10.3390/s21144677

**Published:** 2021-07-08

**Authors:** Razan M. AlZoman, Mohammed J. F. Alenazi

**Affiliations:** 1 Department of Computer Engineering, CCIS, King Saud University, 11451 Riyadh, Saudi Arabia; 441203669@student.ksu.edu.sa; 2 Ministry of Communications and Information Technology, 12382 Riyadh, Saudi Arabia

**Keywords:** machine learning, traffic classification, smart city, quality of service, Internet of things, supervised learning

## Abstract

Smart city networks involve many applications that impose specific Quality of Service (QoS) requirements, thus representing a challenging scenario for network management. Solutions aiming to guarantee QoS support have not been deployed in large-scale networks. Traffic classification is a mechanism used to manage different aspects, including QoS requirements. However, conventional traffic classification methods, such as the port-based method, are inefficient because of their inability to handle dynamic port allocation and encryption. Traffic classification using machine learning has gained research interest as an alternative method to achieve high performance. In fact, machine learning embeds intelligence into network functions, thus improving network management. In this study, we apply machine learning algorithms to predict network traffic classification. We apply four supervised learning algorithms: support vector machine, random forest, *k*-nearest neighbors, and decision tree. We also apply a port-based method of traffic classification based on applications’ popular assigned port numbers. Then, we compare the results of this method to those obtained from the machine learning algorithms. The evaluation results indicate that the decision tree algorithm provides the highest average accuracy among the evaluated algorithms, at 99.18%. Moreover, network traffic classification using machine learning provides more accurate results and higher performance than the port-based method.

## 1. Introduction and Motivations

**Figure 1:**
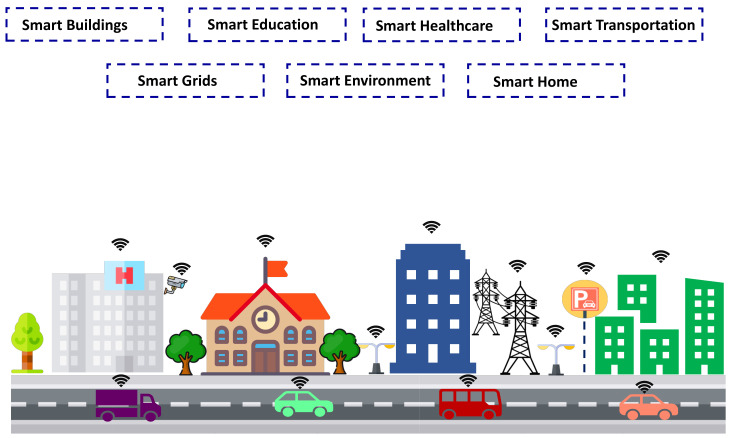
Typical architecture of a smart city network.

The Internet of Things (IoT) is a technological revolution that has gained importance over time. Its substantial impact on many aspects of daily living is the strength of the IoT [1]. In addition, IoT technology plays a vital role in economic growth. Therefore, technology companies and research centers invest in the development and research of IoT solutions [2]. The IoT applications interconnect a variety of objects, such as actuators, sensors, smart devices, and smart home appliances, to the Internet in order to send and process their data [3,4]. Thus, IoT technology is essential to enable smart city services. A smart city is a paradigm that exploits the recent evolution of communication technologies to improve the services provided to its inhabitants and their quality of life [5,6]. It involves many smart solutions [7], such as smart buildings, smart education, smart healthcare, smart transportation, smart grids, smart environments, and smart homes (Figure 1), which can benefit citizens by facilitating smart services. Nevertheless, such applications have diverse and specific requirements that increase the challenges and complexity of network management.

Different applications consist of multiple objects (e.g., sensors, actuators), generate massive network traffic, and have different Quality of Service (QoS) requirements, such as bandwidth, loss, delay, jitter (variation in delay), and best-effort options [8]. For instance, video surveillance supports readiness and response for traffic emergencies and accidents or localization of busy roads [9]. This application has stringent requirements, including high bandwidth and low jitter, for the network traffic to reach its destination. Real-time applications, such as online gaming and telephony, require guaranteed correct interactions and are highly sensitive to delay. The immense expansion of these smart city applications with different traffic types causes challenges to the QoS support because of their diversity. Thus, these challenges should be properly addressed.

Supporting QoS requirements was not an initial design goal of internet architecture; internet architecture was intended for best-effort data delivery. However, several efforts have been devoted to satisfying QoS requirements, including Integrated Services and Differentiated Services [10]. Integrated Services aim at both multicast and unicast applications and offer a QoS guarantee per flow by reserving sufficient network resources along the path, where each router stores an internal state per flow. Hence, Integrated Services increase complexity in routers that are more susceptible to crashes. Moreover, they undermine network scalability through multiple flows because the state of the flows at each node should be stored [8,11,12]. In contrast, Differentiated Services aim to improve the scalability problem of Integrated Services. They group traffic flows into QoS classes using the Differentiated Services Codepoint field in IPv4 and IPv6 headers to satisfy QoS requirements differently compared with flow-based QoS treatment [8,13]. However, these approaches have not been adopted in large-scale networks [8,14].

Traffic classification is essential for many network applications, such as monitoring, QoS management, and security [15]. Traffic classification can implement a mechanism for service differentiation that classifies the traffic flow according to the application type (e.g., streaming, Voice over IP) [16]. Thus, resources can be allocated based on application requirements, such as bandwidth and delay, thus guaranteeing QoS support. Several methods are available for traffic classification without modifying the TCP/IP header. The port-based method classifies the traffic based on the assigned port numbers [17]. However, recent applications use dynamic ports and tunneling, thus rendering this technique ineffective. Alternatively, deep packet inspection classifies traffic according to the packet payload, which is matched with a set of predefined signatures [18]. Nevertheless, this method poses privacy challenges, cannot handle encrypted data, and is computationally expensive [19,20,21].

**Figure 2:**
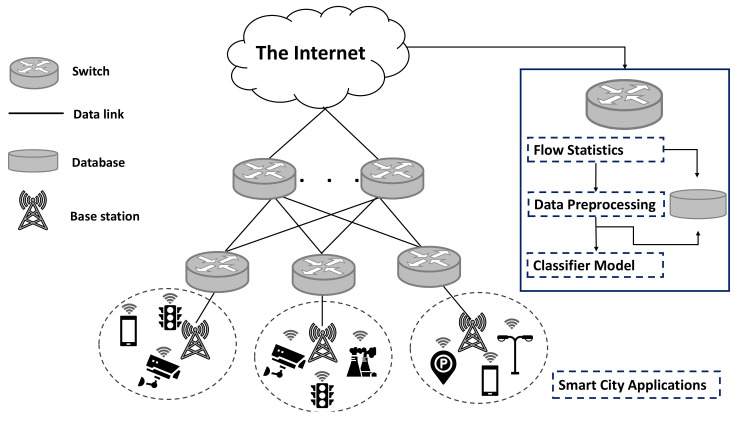
Typical scenario illustrating traffic classification based on machine learning in a smart city network.

Traffic classification based on machine learning algorithms has attracted research interest in view of its expected accuracy and efficiency. Machine learning algorithms involve various steps when adopting supervised learning. First, traffic features that represent the attributes of the flows (e.g., packet length) are identified. Second, the machine learning model is constructed. Third, the classifier is trained to associate specific features with known traffic classes. Finally, the model is applied to classify data traffic, predicting the classes in traffic flow. A typical scenario of traffic classification based on machine learning to improve the QoS in smart city networks is illustrated in Figure 2.

The contributions of this study are summarized as follows. First, we show that machine learning algorithms can accurately classify and predict network traffic. We compare four supervised machine learning algorithms and evaluate their performance for traffic classification, establishing the effectiveness of statistical features. Second, we perform port-based traffic classification based on port numbers to distinguish services running over a network. Finally, we evaluate the performance of the port-based method in comparison with the machine learning algorithms. Our results demonstrate that the decision tree (DT) algorithm provides the highest average accuracy (99.18%) among the evaluated machine learning algorithms, whereas the *k*-nearest neighbors (KNN) algorithm provides the lowest accuracy (97.16%). Overall, traffic classification using machine learning algorithms is more accurate than that using the port-based method. Machine learning algorithms leverage various statistical features in addition to the port number for classification, whereas the port-based method relies solely on assigned port numbers for different applications, which provides ineffective results as many services use dynamic or a variety of ports over a network.

The remainder of this paper is organized as follows: A brief background of machine learning algorithms is presented in Section 2. The related work is discussed in Section 3. The proposed traffic classification method, based on machine learning, is detailed in Section 4. In Section 5, we present the performance evaluation, including the dataset, performance measures, and experimental setup. Section 6 reports the evaluation results. Finally, Section 7 concludes the paper and presents directions for future studies.

## 2. Background

In this section, we describe machine learning algorithms with supervised and unsupervised learning.

### 2.1. Supervised Learning Algorithms

Supervised learning provides knowledge about new samples based on predefined labels. The corresponding machine learning model is trained by a set of inputs with known outputs. This type of learning allows the model to examine features and then create the relations to predict the class label of previously unseen samples. Supervised learning is used to build a system that predicts an output from a given input, using previously learned rules [17]. There are two main types of supervised machine learning problems: classification and regression. In classification, the model predicts the class label, which is a predefined categorical output. In regression, a continuous output is predicted. Supervised learning involves training and testing. During training, the classifier model is built to examine the provided dataset. During testing, the classifier model automatically assigns the learned classes to a test dataset containing previously unseen samples [22,23]. Common supervised learning algorithms include random forest (RF), KNN, DT, neural network, and support vector machine (SVM) [24].

### 2.2. Unsupervised Learning Algorithms

In unsupervised learning, an unlabeled input is provided to the machine learning algorithm. Thus, the output for a sample is not defined. Unsupervised learning is implemented without guidance and aims to find a pattern or structure in input data to group samples based on the similarity or statistical relations between features [18]. Each group with the same pattern obtained from the input data is called a cluster. The model examines and clusters patterns but cannot evaluate the correctness of the results. Unsupervised learning allows new clusters to be determined [22] using algorithms such as *k*-means clustering and self-organizing maps [24].

## 3. Related Work

This section presents related work from the literature and a discussion of their different approaches. Some studies have shown a comparative analysis of traffic classification based on machine learning utilizing different datasets, such as the backbone network, while others have used machine learning for traffic classification or investigated QoS support for smart city applications across different layers, such as the data link layer and transport layer. For instance, Aureli et al. [25] proposed a dynamic classification method called learning-based Differentiated Services to discover traffic characteristics and dynamically assign service classes to IP packets. They applied machine learning methods (e.g., linear discriminant analysis, *k*-means clustering) considering packet characteristics such as the unbalanced traffic distribution between classes. Their proposed method adjusted the classification results dynamically. Although our approach and that of the authors’ share the same objective, which is to classify traffic, the authors applied semisupervised techniques to generate a different number of subclasses from the Differentiated Services labels. However, in our approach, we apply four supervised machine learning algorithms to classify network traffic, using 11 classes.

Zhongsheng et al. [26] proposed an SVM to classify network traffic in campus backbone networks. They applied the SVM to traffic classification through data collection and feature generation. The SVM achieved reliable and accurate results, reaching 99.31% and 96.12% accuracy using biased and unbiased test samples, respectively. However, they only analyzed the SVM, neglecting other machine learning algorithms because algorithm accuracy is not always the most required objective. In fact, real-time applications are more sensitive to delay than to accuracy. Therefore, the execution time of different machine learning algorithms has to be considered.

Al-Turjman [27] handled the wireless medium access problem under rapid mobility in smart cities. The resulting framework uses LTE (Long Term Evolution), while improving the QoS of mobile applications. In addition, it minimizes the delay and error in real-time smart transportation. The framework integrates a Markovian process into the IEEE 802.16 standard to investigate various QoS measures, such as the average packet delay. Moreover, a design for mobile vehicular cloud is proposed considering multiple conditions, such as traffic and weather. The design uses the cellular infrastructure to stream data and video but does not consider machine learning techniques that might provide better and more-efficient decisions.

Yao et al. [28] proposed a traffic classification method mainly intended for smart city networks. Their method relies on deep learning (DL), using a capsule network model for efficient classification. The proposed method aims to remove the manual selection of network traffic features. While this method uses only an improved convolutional neural network model to enhance the feature selection, we rely on four supervised machine learning algorithms and compare their results for traffic classification, aiming to improve the QoS in smart city networks by classifying the network traffic.

Miao et al. [29] compared six machine learning algorithms for traffic classification: Naive Bayes, RF, SVM, H${}_{2}$O, KNN, and DT. They used principal component analysis for feature extraction and analyzed its influence on the classification results. Experimental results showed that RF and KNN were the top performing algorithms overall. Without principal component analysis, the accuracy was 92.92% and 84.56% for RF and KNN, respectively. In contrast, our traffic classification algorithms achieved higher accuracy, reaching 99.08% and 97.16% for RF and KNN, respectively. Although our datasets contain campus data traffic and their datasets contain ISP data traffic, they are both considered backbone network traffic types. Therefore, they share similar data traffic.

Perera et al. [30] compared six supervised learning algorithms for traffic classification: Naive Bayes, Bayesian network, RF, DT, Naive Bayes tree, and multilayer perceptron. Experiments were conducted using two feature selection methods and five traffic classes. The results showed that the RF and DT algorithms provided the highest classification accuracy, with 96% and 95% average accuracy, respectively. However, our traffic classification algorithms achieved superior performance, with 99.08% and 99.18% average accuracy for RF and DT, respectively.

Rahman et al. [31] proposed a cloud robotics framework that is suitable for smart city applications. In the framework, a robotic agent leverages cloud services through task offloading to improve the QoS and system performance. An optimization problem is formulated for a directed acyclic graph, and a genetic algorithm determines the optimal offloading decisions and solves the optimization problem. Unlike this development, we improve the QoS in smart city networks by adopting traffic classification based on machine learning.

To summarize, machine learning algorithms have been used to compare classifier performance considering supervised algorithms. In addition, deep learning techniques have been studied and various methods have been proposed to improve QoS in smart city networks. Unlike existing studies, we provide a comprehensive study and evaluate the performance of supervised classification algorithms—namely, SVM, RF, KNN, and DT—to improve the QoS in smart city networks and classify network traffic according to statistical features. Moreover, we design and implement a port-based traffic classification method for comparison with the machine learning algorithms.

## 4. Traffic Classification Method Based on Machine Learning

**Figure 3:**
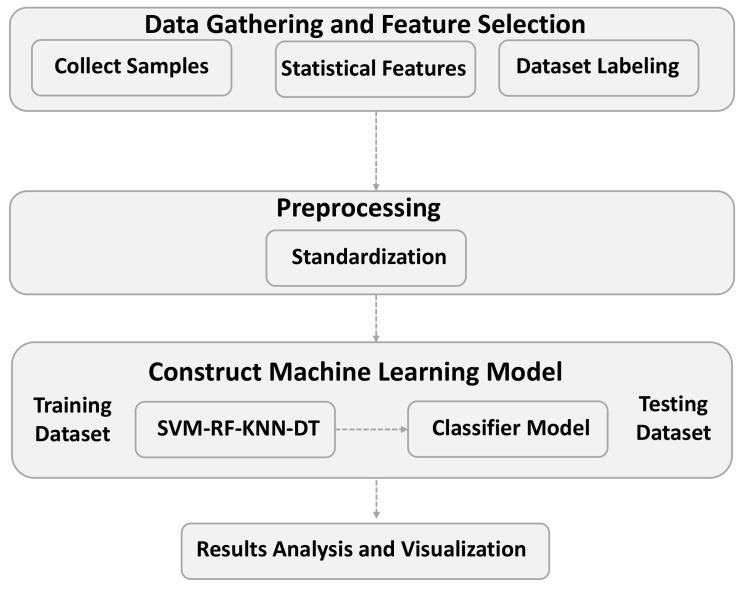
Steps to build and evaluate proposed machine learning algorithms.

We adopt a four-step method for traffic classification based on machine learning (Figure 3): data gathering and feature selection; preprocessing; construction of a machine learning model; result analysis and visualization. In data gathering and feature selection, traffic flow samples are collected into a dataset for evaluation. The collected dataset is described in Section 5. We then remove nonstatistical features and manually label each sample with its corresponding class to create the training and test datasets. During preprocessing, the features are scaled by applying a standardization method to the samples. Then, the machine learning algorithms use the training dataset to determine the model for traffic classification. We compare four common supervised machine learning algorithms: SVM, RF, KNN, and DT. Then, the test dataset is used to evaluate each algorithm. During analysis, we use four measures to evaluate the algorithm performance: accuracy, precision, recall, and F1-score. In addition, we adopt *k*-fold cross-validation to evaluate the classification performance.

## 5. Evaluation of Traffic Classification

In this section, we describe the evaluation environment used in this study. We detail the dataset and its traffic flows. Next, we introduce the performance measures to evaluate the model. Finally, we present the experimental setup to evaluate the port-based method and machine learning algorithms.

### 5.1. Dataset

We use the dataset constructed by Moore and Zuev [32] to apply the machine learning algorithms and port-based method to traffic classification. The dataset has related smart city data traffic characteristics, such as diversity in data sources, high traffic samples, and various data types. The dataset was collected in a computer laboratory at Cambridge University. It comprises 10 datasets monitored at different times of the day from one Internet website, which hosted around 1000 users connected to the Internet via a full-duplex gigabit link. The dataset consists of 248 features, such as flow duration, TCP port number, and packet interarrival time mean and variance. The dataset contains around 377,000 samples based on TCP traffic flows. The classes with their corresponding applications are listed in Table 1. We collect random samples from each class to construct a new dataset and remove some features, including those representing nonstatistical information. In addition, we remove the class Games, which has few samples. As a result, we obtain the 11 classes listed in Table 2.

### 5.2. Performance Measures

To evaluate the performance of the machine learning algorithms and port-based method, we consider various measures: accuracy, precision, recall, and F1-score. The accuracy is the ratio of correctly classified traffic flow samples to the total number of samples (Equation (1)):

(1) $$Accuracy=\frac{TP+TN}{TP+TN+FP+FN}$$

where the true positives (*TP*) represent the number of traffic flows correctly classified into the class they belong to, and the true negatives (*TN*) represent the number of traffic flows correctly classified as not corresponding to a class. In addition, the false positives (*FP*) represent the number of traffic flows incorrectly classified into a class, and the false negatives (*FN*) represent the number of traffic flows incorrectly classified as not belonging to a class. We use the average of tenfold cross-validation to measure the accuracy and improve the reliability of the results.

In some cases, when the dataset has a class representing the majority of sample values, the accuracy score value might not precisely reflect the classifier model’s performance. To avoid this problem, we also use other performance measures (precision, recall, and F1-score) to present the performance of the classifier model. The precision is a measure of the ratio of positive, correctly predicted traffic classes to the total number of positive classification predictions (Equation (2)):

(2) $$Precision=\frac{TP}{TP+FP}$$

The recall measures the ratio of the actual positive, correctly predicted traffic classes (Equation (3)):

(3) $$Recall=\frac{TP}{TP+FN}$$

The F1-score measures the average of precision and recall (Equation (4)):

(4) $$F1=\frac{2\times Precision\times Recall}{Precision+Recall}$$

### 5.3. Experimental Setup

All the experiments for the machine learning algorithms and port-based method were implemented in Python. Specifically, we used the Scikit-learn library to implement, train, and test the machine learning algorithms. We split the dataset into training and test datasets, containing 75% of the samples for training and 25% of the samples for testing. We also performed data preprocessing, including feature scaling using standardization for the feature’s sample values to reflect the same properties and avoid any bias towards a specific feature. The objective of the standardization technique is to rescale the feature’s sample values with a mean and standard deviation of 0 and 1, respectively. The standardized value score (*z*) of a sample is calculated using Equation (5):

(5) $$z=\frac{x-\mu }{\sigma }$$

where *x* is the sample value to be standardized, *μ* is the mean of the training samples, and *σ* represents the standard deviation of training the samples.

In addition, we set different parameters in the machine learning algorithms to promote accuracy. These parameters are related to each machine learning algorithm. To evaluate the classification performance and prevent overfitting, we used tenfold cross-validation to measure the accuracy, which provides reliable results when applied to machine learning algorithms. To apply and evaluate the port-based method, we determined a list of popular and well-known port numbers corresponding to the applications included in the dataset.

## 6. Results and Discussion

This section presents the evaluation results obtained from the machine learning algorithms and the port-based method for traffic classification. We present the evaluation of the machine learning algorithms in terms of performance measures, the impact of the number of classes on accuracy, and their training and execution times. Then, we compare the algorithms with the port-based method.

### 6.1. Evaluation of Machine Learning Algorithms

**Figure 4:**
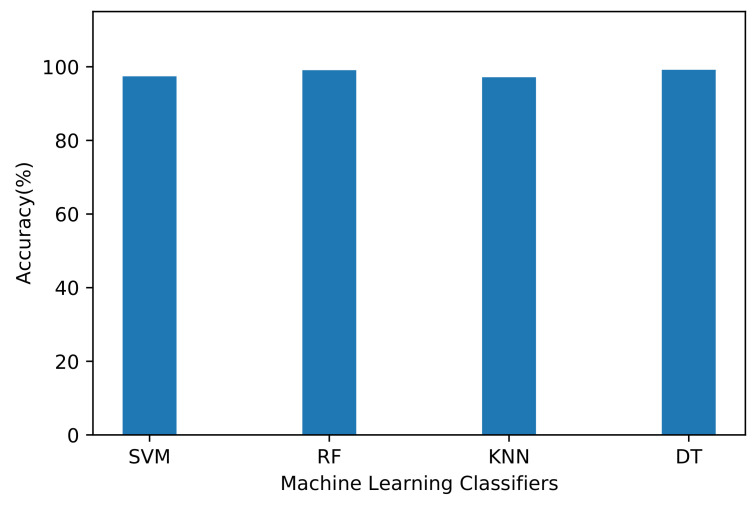
Average accuracy of machine learning algorithms.

We implement and compare four machine learning algorithms: SVM, RF, KNN, and DT. We set different model parameters for each algorithm to improve accuracy. In the SVM implementation, we set a linear kernel. The SVM performs supervised learning and represents the data samples in a high-dimensional space. This space determines a hyperplane to optimally separate the samples and maximize the margin between classes, using support vectors [33]. The average accuracy of SVM reached 97.41%, demonstrating its high performance (Figure 4). The precision, recall, and F1-score per traffic class using SVM are listed in Table 3. The results demonstrate that Interactive and Multimedia classes have the lowest measurements among the classification labels. Class Interactive has a precision of 0.82, recall of 0.72, and F1-score of 0.77; class Multimedia has a precision of 0.62, recall of 0.83, and F1-score of 0.71. Thus, the number of data traffic samples affects the classification performance, because these two classes have fewer samples than the others.

The RF algorithm creates DTs trained on the data flows and then aggregates the different results to predict the class. In the RF implementation, we consider 50 trees to maintain reasonable execution time and accuracy. We also set entropy as a parameter that yields the best results. The average classification accuracy of RF reached 99.08% (Figure 4), which is a remarkable performance. The evaluation results were also better for classes Interactive and Multimedia. Class Interactive has a precision of 1.00, recall of 0.92, and F1-score of 0.96; class Multimedia has a precision of 0.86, recall of 1.00, and F1-score of 0.92. Table 4 shows that the RF implementation with its many DTs improves class prediction.

**Figure 5:**
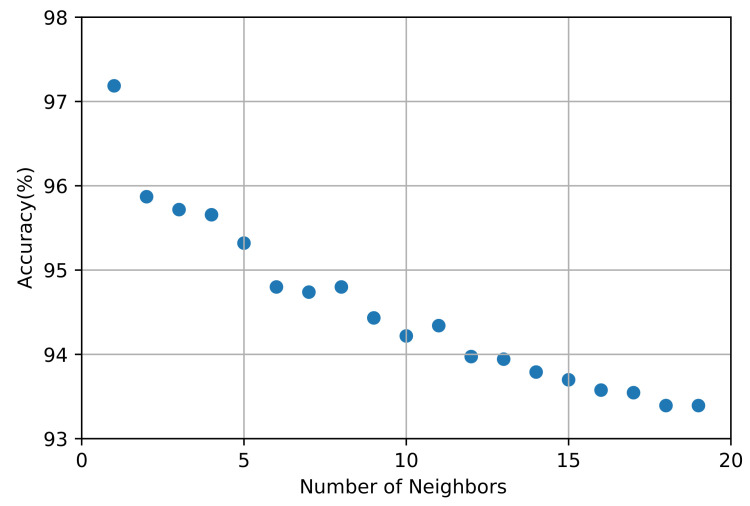
Accuracy according to number of neighbors for *k*-nearest neighbors (KNN) algorithm.

In the KNN algorithm, we use the Manhattan distance that provides high accuracy and performance. The KNN algorithm [34] classifies data by finding the closest *k* neighboring data points. The data class prediction is then based on majority voting between the neighbors according to distance. The type of distance and value of *k* determine the performance of the KNN algorithm. We obtain the best value of *k* from the accuracy obtained for *k* that ranged from 1 to 20, as depicted in Figure 5. The results indicate that the accuracy dropped while increasing the number of neighbors, and a *k* value of 4 yields an accurate classification. The average accuracy of the KNN algorithm reached 97.16%, which is lower than that of other algorithms (Figure ). The precision, recall, and F1-score per class obtained from the KNN algorithm are listed in Table 5.

The DT algorithm consists of multiple nodes and conditions until reaching its leaves to predict classes. In the DT implementation, we use the entropy metric for improved performance. The average accuracy of the DT algorithm reached 99.18% (Figure 4), which is the highest accuracy among the evaluated classification algorithms. The precision, recall, and F1-score per class obtained from the DT algorithm are listed in Table 6, showing high measured values for most classes.

**Figure 6:**
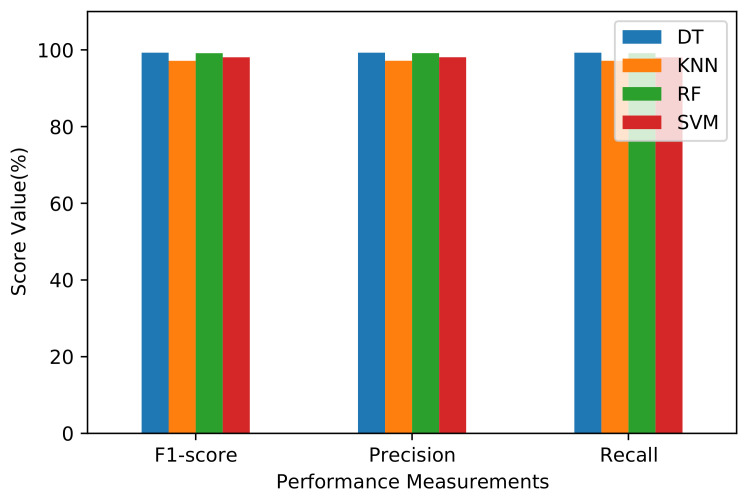
F1-score, precision, and recall performance measurements of machine learning algorithms.

The results of F1-score, precision, and recall for each machine learning algorithm are shown in Figure 6. The results show that for F1-score measurement, the DT algorithm outperforms other algorithms, with a score of 99.27%; the KNN reached 97.15%, the RF reached 99.14%, and the SVM reached 98.07%. For precision, the DT achieved the highest performance with 99.27%, while the KNN reached 97.16%, the RF reached 99.15%, and SVM achieved a score of 98.08%. For Recall, the DT algorithm shows its effectiveness with a score of 99.27%, while the KNN reached 97.16%, the RF reached 99.14%, and SVM achieved 98.07%. Based on these results, the DT algorithm outperforms the other machine learning algorithms in all calculated performance metrics.

**Figure 7:**
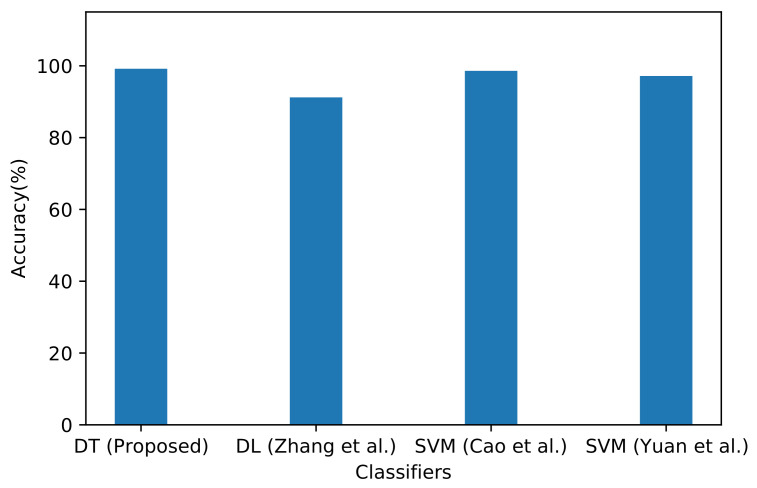
Accuracy comparison between the proposed DT algorithm, the DL method (Zhang et al.) in [35], SVM algorithm (Cao et al.) in [36], and SVM algorithm (Yuan et al.) in [37].

Here, we compare the results of the DT algorithm against other studies with similar approaches. For instance, Zhang et al. [35] proposed a method relying on the DL method with five hidden layers and 10 hidden nodes targeting the same dataset. As shown in Figure 7, our proposed model achieved higher performance results than the DL method, reaching 99.18% accuracy compared with 91.21%. Moreover, the proposed algorithm outperformed Cao et al.’s [36] SVM algorithm targeting the same dataset, which scored 98.6%. Another performance study was proposed by Yuan et al. [37] based on SVM, utilizing the same dataset. The results of unbiased samples achieved a score of 97.17%. However, our proposed DT algorithm achieved higher performance (Figure 7).

Overall, the proposed model of the DT algorithm achieves the highest average accuracy (99.18%) among the evaluated algorithms, whereas the KNN algorithm provides the lowest average accuracy (97.16%). Moreover, the DT algorithm outperforms other machine learning algorithms when it comes to precision and recall evaluation. The results indicate the effectiveness of the model to predict positive traffic classes correctly. In addition, the DT and RF algorithms provide suitable performance for traffic classification.

### 6.2. Impact of the Number of Classes on the Accuracy

**Figure 8:**
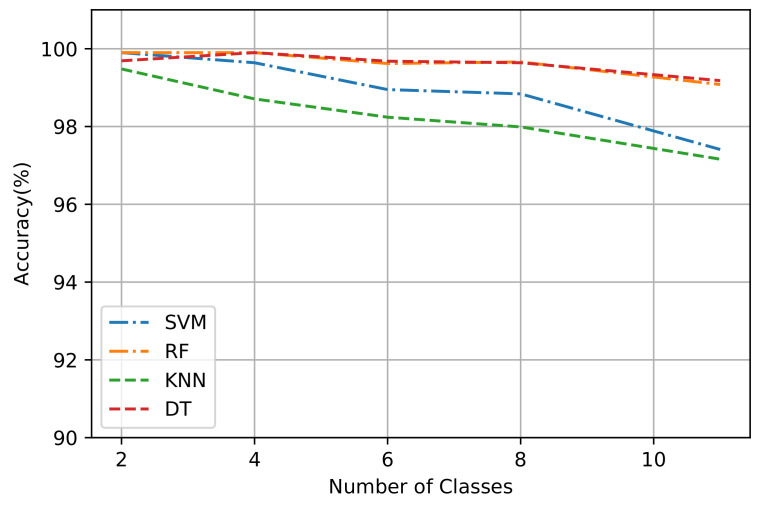
Illustrating the impact of increasing the number of classes on the average accuracy.

This section studies the impact of increasing the number of classes on the accuracy of machine learning algorithms. We assume different numbers of class labels as follows: two, four, six, eight, and eleven. Figure 8 shows the impact of increasing the number of classes on the machine learning algorithms’ accuracy. The results demonstrate that as the number of classes increases, the average accuracy decreases. Furthermore, we observe that the decrease in average accuracy for the DT and RF algorithms is slight compared to the other algorithms, showing their strength for traffic classification. DT starts at 99.69% when the number of classes is 2, then ends at 99.28% when the number of classes is 11. RF starts at 99.90% when the number of classes is 2, then ends at 99.08% when the number of classes is 11. However, in the KNN algorithm, increasing the number of classes drops the average accuracy dramatically; it starts at 99.48% when the number of classes is 2, then ends at 97.16% when the number of classes is 11. This indicates that KNN, which depends on the distance to measure the similarity to the *k*-nearest neighbor’s point, is highly impacted by increasing the number of classes. The SVM algorithm also shows a drop, with an average accuracy of 99.9% and 97.41% in cases of 2 and 11 classes, respectively. By observing these impacts, we conclude that DT and RF yield better performance results than other algorithms in terms of average accuracy when varying the number of classes.

### 6.3. Training and Execution Times

**Figure 9:**
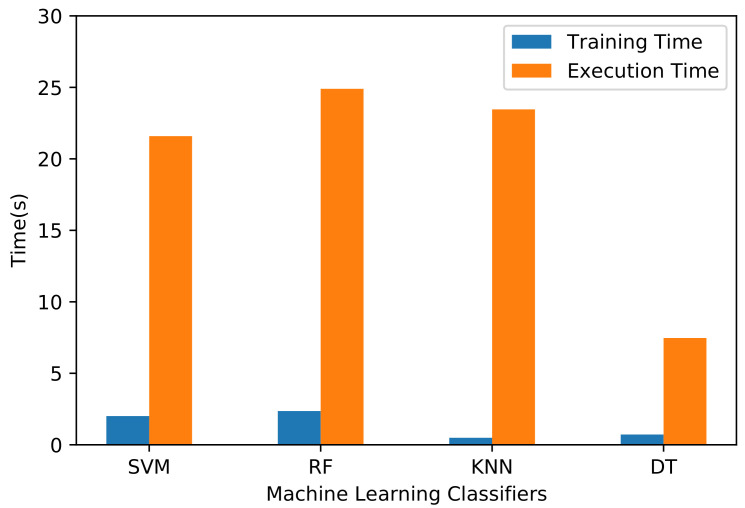
Training and execution times of machine learning algorithms.

Performance evaluation, in addition to accuracy, is essential to characterize machine learning algorithms. In particular, the training and execution times are important performance indicators. The training time is the time taken by a model to train on a dataset, and the execution time represents the total time taken for computations, including data splitting, data preprocessing, and model evaluation. The training and execution times for all the machine learning algorithms are shown in Figure 9. The training and execution times of the SVM algorithm are 2.01 and 21.59 s, respectively. For the RF algorithm, the respective times are 2.36 and 24.90 s. Thus, RF is the slowest algorithm regarding both training and execution times, because it builds and computes several DTs. In contrast, the training time of the KNN algorithm is only 0.49 s because it does not create any model during training but only stores training data for subsequent classification. Nevertheless, its execution time is 23.46 s. This indicates that KNN takes more time to measure the distance to the *k*-nearest neighbor’s data point. The training and execution times of the DT algorithm are 0.72 and 7.47 s. Thus, DT is the fastest among the evaluated algorithms in terms of both training and execution times.

### 6.4. Evaluation of Port-Based Method

We also implement a port-based method for traffic classification in Python. The method relies on popular port numbers for different services, and considers only the standard port numbers per service running over network protocols (e.g., TCP, UDP). The port numbers are used to distinguish different services running over the network for management purposes, including QoS and security. We only consider the corresponding well-known port for each application among classes defined in the dataset (depicted in Table 1). For instance, we define port number 22 for SSH (Secure Shell) services and port 21 for FTP (File Transfer Protocol) applications.

**Figure 10:**
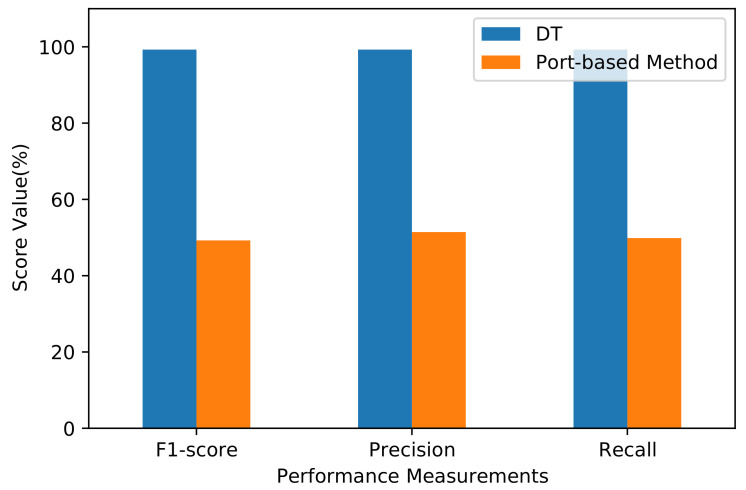
F1-score, precision, and recall performance measurements of DT algorithm and Port-based Method.

We apply the port-based method [38] that classifies the flow from data samples according to the port number. Algorithm 1 shows the pseudocode of the port-based method’s implementation. After applying the method, classification accuracy reached 49.86%, which represents a low value. The results per class are listed in Table 7. The precision, recall, and F1-score in most classes are zero, indicating that the port-based method relies only on popular port numbers to classify data flows. However, the applications in the dataset use dynamic port numbers for many purposes, including security. Some classes, such as Interactive and Mail, show precision, recall, and F1-score values of 1 because the corresponding applications use specific ports when constructing the dataset. However, the port-based method is expected to fail in classes such as Attack, because the port numbers are unknown beforehand, rendering the port-based method ineffective. For comparison, the F1-score, precision, and recall are calculated. Compared to DT, which shows the highest results discussed in Section 6.1, the port-based method reached 49.25% for F1-score, 51.44% for precision, and 49.86% for recall (Figure 10). Overall, the port-based method shows a lower precision, recall, and F1-score than the machine learning algorithms; the latter seem promising because they consider various statistical features in addition to port numbers for traffic classification.
**Algorithm 1:** Port-based Method
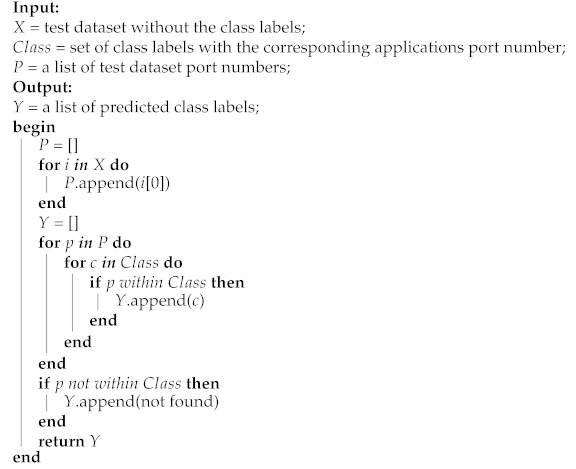


### 6.5. Evaluation Summary

This section summarizes the evaluation results of machine learning algorithms and the port-based method. By observing the results, we conclude that the DT algorithm showed the best average accuracy and execution time performance. This result is considered the best option for network traffic classification. However, smart city applications have different QoS requirements. Therefore, the DT algorithm is suitable for applications that require real-time interactions and are sensitive to delay, such as online games, voice over IP, and streaming applications. For mission-critical services that focus on accuracy rather than execution time, such as intrusion detection systems, the DT and RF algorithms are preferable for network traffic classification. Moreover, we observe that KNN is considered time-consuming, rendering the KNN algorithm inefficient for applications that require rapid decisions. Alternatively, the KNN is preferable for solutions that tend to find similarities between instances. However, machine learning algorithms outperform the port-based method in terms of traffic classification efficiency.

## 7. Conclusions and Future Work

Smart cities are becoming increasingly popular over time. The deployment of smart solutions aims to make our daily lives more comfortable, productive, and efficient. Smart cities involve various applications, data diversity, and a variety of QoS requirements that represent challenges for traffic management. Traffic classification can be used to manage several network aspects, including QoS support. Conventional traffic classification methods, such as the port-based method and deep packet inspection, cannot handle encrypted data and dynamic port numbers. In contrast, machine learning algorithms may solve QoS management and handle complexity. We evaluated four supervised machine learning algorithms for traffic classification: SVM, RF, KNN, and DT. In addition, we evaluated a port-based method for comparison with the machine learning algorithms. The evaluation results demonstrated that statistical features improve traffic classification based on machine learning. The DT algorithm provided the highest average accuracy (99.18%) among the evaluated machine learning algorithms. In contrast, the KNN algorithm provided the lowest average accuracy (97.16%). Moreover, we demonstrated the limited effectiveness of the port-based method for traffic classification, as this method depends on specific port numbers to distinguish network flows. Unlike this method, the machine learning algorithms consider various features, as well as the assigned port number, for traffic classification. In future work, we plan to investigate routing problems by integrating machine learning algorithms for traffic classification into various smart city applications that handle critical data. Moreover, we plan to compare other machine learning algorithms for traffic classification, such as eXtreme Gradient Boosting (XGBoost).

## Figures and Tables

**Table 1:** Applications for traffic classification.

Classification	Application
Bulk	ftp
Database	postgres, sqlnet oracle, ingres
Interactive	ssh, klogin, rlogin, telnet
Mail	imap, pop2/3, smtp
Services	X11, dns, ident, ldap, ntp
WWW	www
P2P	KaZaA, BitTorrent, GnuTella
Attack	Internet worm and virus attacks
Games	Half-Life
Multimedia	Windows Media Player, Real

**Table 2:** Characteristics of the traffic classification dataset used in this study.

Traffic Class	Samples
Attack	1500
Database	1068
FTP–Control	993
FTP–Data	2019
FTP–Passive	1297
Interactive	110
Mail	2081
Multimedia	43
P2P	542
Services	1921
WWW	1499

**Table 3:** Precision, recall, and F1-score per class for SVM classification.

Traffic Class	Precision	Recall	F1-Score
**Attack**	0.95	0.95	0.95
**Database**	1.00	1.00	1.00
**FTP–Control**	0.98	0.99	0.99
**FTP–Data**	0.99	1.00	1.00
**FTP–Passive**	0.98	1.00	0.99
**Interactive**	0.82	0.72	0.77
**Mail**	0.99	0.99	0.99
**Multimedia**	0.62	0.83	0.71
**P2P**	0.92	0.96	0.94
**Services**	1.00	0.99	0.99
**WWW**	0.98	0.95	0.96

**Table 4:** Precision, recall, and F1-score per class for RF classification.

Traffic Class	Precision	Recall	F1-Score
**Attack**	0.98	0.97	0.98
**Database**	1.00	1.00	1.00
**FTP–Control**	1.00	1.00	1.00
**FTP–Data**	0.99	1.00	1.00
**FTP–Passive**	0.98	1.00	0.99
**Interactive**	1.00	0.92	0.96
**Mail**	1.00	1.00	1.00
**Multimedia**	0.86	1.00	0.92
**P2P**	0.97	0.97	0.97
**Services**	1.00	0.99	1.00
**WWW**	0.99	0.97	0.98

**Table 5:** Precision, recall, and F1-score per class for KNN classification.

Traffic Class	Precision	Recall	F1-Score
**Attack**	0.95	0.95	0.95
**Database**	0.97	1.00	0.99
**FTP–Control**	0.96	0.99	0.98
**FTP–Data**	0.99	0.99	0.99
**FTP–Passive**	0.97	0.97	0.97
**Interactive**	0.81	0.68	0.74
**Mail**	0.98	0.98	0.98
**Multimedia**	0.62	0.83	0.71
**P2P**	0.88	0.89	0.89
**Services**	1.00	0.99	0.99
**WWW**	0.97	0.94	0.96

**Table 6:** Precision, recall, and F1-score per class for DT classification.

Traffic Class	Precision	Recall	F1-Score
**Attack**	0.99	0.98	0.99
**Database**	1.00	1.00	1.00
**FTP–Control**	1.00	1.00	1.00
**FTP–Data**	1.00	1.00	1.00
**FTP–Passive**	0.98	0.99	0.99
**Interactive**	1.00	1.00	1.00
**Mail**	0.99	1.00	1.00
**Multimedia**	1.00	1.00	1.00
**P2P**	0.94	0.98	0.96
**Services**	1.00	0.99	1.00
**WWW**	0.99	0.98	0.98

**Table 7:** Precision, recall, and F1-score per class for conventional port-based classification.

Traffic Class	Precision	Recall	F1-Score
**Attack**	0.00	0.00	0.00
**Database**	0.00	0.00	0.00
**FTP–Control**	1.00	0.99	0.99
**FTP–Data**	0.00	0.00	0.00
**FTP–Passive**	0.00	0.00	0.00
**Interactive**	1.00	1.00	1.00
**Mail**	1.00	1.00	1.00
**Multimedia**	0.00	0.10	0.18
**P2P**	1.00	0.99	0.99
**Services**	0.77	0.95	0.85
**WWW**	0.00	0.00	0.00

## Data Availability

Not applicable.
